# Biological Responses of Ceramic Bone Spacers Produced by Green Processing of Additively Manufactured Thin Meshes

**DOI:** 10.3390/ma13112497

**Published:** 2020-05-30

**Authors:** Joaquim Minguella-Canela, Jose Antonio Calero, Feza Korkusuz, Petek Korkusuz, Berna Kankılıç, Elif Bilgiç, M. Antonia De los Santos-López

**Affiliations:** 1 Centre CIM, Departament d’Enginyeria Mecànica, Universitat Politècnica de Catalunya, Av. Diagonal, 647, 08028 Barcelona, Spain; tania.santos@upc.edu; 2 AMES PM Tech Center, Camí de Can Ubach, 8. Pol. Ind. “Les Fallulles”, 08620 Sant Vicenç dels Horts, Spain; jacalero@ames.group; 3 Department of Sports Medicine, Medical Faculty, Hacettepe University, Sihhiye, Ankara 06100, Turkey; feza.korkusuz@gmail.com; 4 Department of Histology and Embryology, Medical Faculty, Hacettepe University, Sihhiye, Ankara 06100, Turkey; petek@hacettepe.edu.tr (P.K.); elif.bilgic@hacettepe.edu.tr (E.B.); 5 Department of Biotechnology, Graduate School of Natural and Applied Sciences, Middle East Technical University, Cankaya, Ankara 06800, Turkey; uysalberna@yahoo.com

**Keywords:** additive manufacturing, implants, bioceramics, porous scaffolds, bone tissue engineering, cell proliferation, biological responses

## Abstract

Bone spacers are exclusively used for replacing the tissue after trauma and/or diseases. Ceramic materials bring positive opportunities to enhance greater osteointegration and performance of implants, yet processing of porous geometries can be challenging. Additive Manufacturing (AM) opens opportunities to grade porosity levels in a part; however, its productivity may be low due to its batch processing approach. The paper studies the biological responses yielded by hydroxyapatite with β-TCP (tricalcium phosphate) ceramic porous bone spacers manufactured by robocasting 2-layer meshes that are rolled in green and sintered. The implants are assessed in vitro and in vivo for their compatibility. Human bone marrow mesenchymal stem cells attached, proliferated and differentiated on the bone spacers produced. Cells on the spacers presented alkaline phosphatase staining, confirming osteogenic differentiation. They also expressed bone-specific COL1A1, BGAP, BSP, and SPP1 genes. The fold change of these genes ranged between 8 to 16 folds compared to controls. When implanted into the subcutaneous tissue of rabbits, they triggered collagen fibre formation and mild fibroblastic proliferation. In conclusion, rolled AM-meshes bone spacers stimulated bone formation in vitro and were biocompatible in vivo. This technology may give the advantage to custom produce spacers at high production rates if industrially upscaled.

## 1. Introduction

Bone tissue, which is an integral part of the musculoskeletal tissue, is a heterogeneous vascular matrix of mainly collagen type 1, cells and calcium phosphate [1]. Its main functions are protection and support of internal organs, structural support of movement, storage of minerals, and synthesis of blood cells. Bone needs replacement when the tissue is lost due to major trauma and/or diseases such as sarcoma. Basic tissue engineering approaches have proposed replacing bone by combining collagen type 1, cells and calcium phosphates (CPs) that are brought into shape to scaffolds, which can then act as a replacement [2]. Maintaining mechanical stability until the spacer is replaced by original tissue is a current challenge. Homeostasis of the host bone is another factor that could cause an immune response to the implanted spacer [3].

Several alternatives can be found for replacing missing bone tissue in living vertebrates. Autografts, the grafts obtained from the patient him/herself, are the first choice of surgeons, but bone harvesting is limited. Donor site morbidity including pain, infection and loss of function is also a major problem. Allografts, the grafts taken from other people, may cause inflammation, transfection and have ethical concerns. Xenografts that are obtained from other species are seldom used in some cases but again the patient faces inflammation [4]. Therefore, there is a clear need for obtaining artificial bone spacers.

The biocompatible materials used in prostheses cover a wide range. Metals are widely utilized, mainly low carbon steels and titanium alloys. Ceramic materials are used as well, such as Zirconium Dioxide (ZrO_2_), aluminium oxide (alumina, Al_2_O_3_), alumina toughened zirconia (ATZ), [5,6,7,8] hydroxyapatite and tricalcium phosphate (TCP) [9,10], calcium aluminates (C3A) and titanium oxides (TiO_2_), among others [11]. In addition, polymers such as Teflon, nylon, silicones, and some others, as well as new material compositions that are tailored developed according to specifications, including nanomaterials, metal-carbon or metal-nitrogen ceramics, and other intermetallic alloys are used [12]. Comparing the different properties for interacting with the environment of the materials used, the ceramic family is the one that can enhance the highest biocompatibility with bones in terms of absorbability and bioactivity. Various calcium spacers of calcium sulphate, calcium phosphate and hydroxyapatite including nano-forms as pure or composites have been used to establish appropriate spacers [1,13]. However, the mechanical properties they provide are the lowest within the different choices available, due to their low elasticity and high density.

Related to manufacturing, one of the biggest setbacks of the ceramic implant production is the difficulty of these materials to be machined, specifically due to its fragility, brittleness, and abrasive behaviour, as previously encountered by the authors [14,15]. To this regard, Additive Manufacturing (AM) recently allowed the precise production of bone spacers with sufficient mechanical strength for handling and in combination with non-organic and/or organic components. However, AM procedures suffer from their innate additive and batch processing ways, which results in very low production rates [16]. In this sense, some research should be conducted to find combinations of AM processes with specific post-processing to speed up the materialization of ceramic implant units.

Current studies focus on the simplification of the ink’s formulations, on the use of biocompatible additives, on the design of structures capable of maximizing the mechanical behaviour of the materials used, and on the effects of the post-processing activities related to the completion of the implants. Nevertheless, when considering the industrial scaling of the process, it should be addressed how to convert batch processes into more scalable, mass producing, economically favourable processes that could be capable to meet the quality requirements set for real clinical application.

In particular, it is important to bear in mind that in most of the 3D printing processes, the relative displacements of the nozzle head in the normal direction to the printing plane (the ‘z’ axis if the paths are laid on the ‘x’-‘y’ plane), usually account for the biggest time losses in the form of air time. Therefore, one step beyond on the scalability of the implants could be, not using the AM capabilities for printing a tall 3D structure, but to print 2D green patterns (fabric-like meshes) that could be then postprocessed in physical geometrical operation (rolling, bending, folding) to generate cylinders, cuboids or some other structures. In this approach, the initial thin green flat ceramic mesh could be obtained in a fast and continuous manner. Especially if it is possible for postprocessing operations to be automated, this process variation could trigger the output maximisation of 3D printed ceramic implants.

In this context, the present study focuses on evaluating the biological responses yield by bioceramic bone spacers produced in a new production method that could be industrially scaled up, achieving high productivity. The raw material utilised was a compound of hydroxyapatite with β-TCP produced on purpose with a ratio of 60/40 (wt%). The production method consists in manufacturing cylindrical spacers in the ceramic compound by the application of robocasting, to obtain a flat mesh, and a rolling operation (in green) to achieve the desired spacer geometry. The method finishes with a debinding and a sintering step to eliminate the organic part and to consolidate the structure. With the samples produced by this method, an exhaustive in-vivo and in-vitro characterization is conducted for the ceramic implants, utilizing techniques for evaluating cell adhesion, proliferation, osteogenic differentiation, osteogenic differentiation, bone-specific gene modulation, and in vivo response of these spacers in order to quantify the biological performance of the parts obtained. The feasibility to obtain the samples, the biological responses obtained and the quantification of the process parameters of a conceptual scaled-up solution give significance to the industrial viability of the new process envisaged.

### 1.1. Implants in Use: The Case of the Bone Spacers

Bone spacers are generally used during revision surgery to control postoperative infection as temporary implants [17]. Poly-methyl-methacrylate (PMMA) cement is the most known material used as a bone spacer [18]. Unfortunately, PMMA has several drawbacks; namely, PMMA is a nonbiodegradable polymer, so a second surgery is needed for its removal from the body and also the polymer shows an exothermic reaction during polymerization; thus, only heat stable antibiotics can be carried and the material has a poor elution profile [19,20].

Besides control of infection, spacers are also used for critical bone defects. To accomplish this, Masquelet technique was developed [21]. The technique depends on implanting PMMA spacer to defect the site for triggering biological membrane formation. After explantation of the spacer, the space was replaced with a graft without damaging the induced membrane [22].

The two main characteristics that are fundamental for the materials to be used in traumatology implants and prostheses are (i) biocompatibility and (ii) best fit with the mechanical properties of the bones in the implant context [23]. To characterize the bone properties, biocompatible, 3D shaped, porous biomaterials that have the same biomechanical properties with the bone are also favoured in bone tissue engineering for bone defects.

The bones are structured in light yet flexible structural solutions, which adapt to the functions required by the organism, achieving the maximum efficiency with the minimum weight. In general, and classifying the bones depending on their mechanical behaviour [24,25], the trabecular bone, in the form of long bones with mineral compounds, has lower elastic modulus, density and yield strength than cortical bones, as well as a more dense structure that forms the outer surface of the bone [26].

The materials utilized in implants can interact with the host tissue in different ways. Depending on their manner of interaction, the materials used can be classified as bioinert, bioresorbable or bioactive [27].

### 1.2. Additive Manufacturing for the Fabrication of Implants

AM is a rapidly developing group of manufacturing means based on layer-by-layer material production from a computerized 3D model [28]. Typical available materials include thermoplastics (polyamides, Acrylonitrile Butadiene Styrene, polycarbonates, elastomers, etc.), photocurable polymers, metals, and alloys (steel, titanium and aluminium). Also, some ceramics materials are available, although it is an emergent group that requires further study and analysis. Controlling the grid structure is key, and modelling is being analysed, finding analytical expressions of the pore sizes obtained as a function of parameters such as infill percentage, nozzle diameter and layer height [29].

Robocasting is an AM technique capable of achieving ceramic porous parts by extruding horizontal layers of ceramic inks, similarly to the Fused Deposition Modelling (FDM) process, but from a syringe or similar dispensing systems [30]. In this case, the inks need to meet suitable viscoelastic properties to be extruded through small needle heads. Once extruded, the inks must be able to maintain the shape and to support the following layers of material to be placed on top of the previous ones [31].

**Figure 1:**
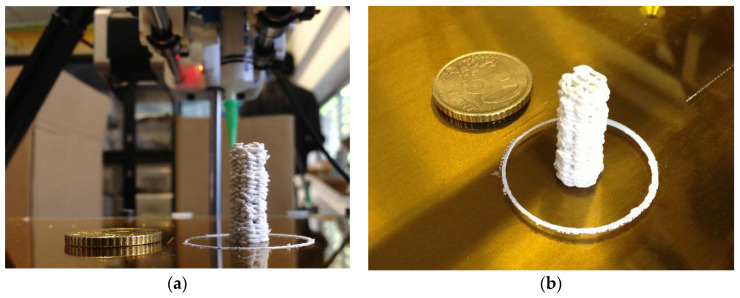
Ceramic ink column constructed adding 40 circular horizontal layers of extruded material with a Robocasting syringe 3D printer (Fundació Privada Centre CIM, Barcelona, Spain) at Centre CIM: (**a**) Elevation view; (**b**) Isometric view.

At the present time, there exist inks composed of hydroxyapatite, β-TCP and bioactive glasses, which are suitable for robocasting, with average particle sizes between 1 and 3 µm. In this regard, the control of the morphology, specific surface and reactivity of the materials are important parameters to optimize the performance of the process. With these types of inks, some authors in the literature achieved the deposition of filaments of 250 µm of diameter [32,33]. Some previous experiences (see Figure 1a,b) demonstrated the feasibility to obtain tall green structures (composed by tenths of horizontal layers of diameters ranging between 150–410 µm), with controlled porosity derived from the application of specific computer-controlled patterns via automatic 3D printing robocasting [34]. With the use of inks based on bioactive glasses, some authors have achieved 3D print filaments with diameters of 30 µm [35].

Lithographic processes have proven to be capable of manufacturing complex ceramic implants [36]. Lithography-based Ceramic Manufacturing (LCM) is a technology capable of producing complex ceramic parts by consolidating the photopolymer in a suspension by the action of light. It is a fast processing method for obtaining dense, strong, and precise complex three-dimensional ceramic parts [37]. LCM can achieve high processing speeds in wide structures because the light can actuate polymerizing all the material contained in a layer at the same time. Same as in robocasting, ceramic parts produced are in green and must undergo drying, debinding and sintering operations before achieving a solid internal structure. The process works in batches of the number of parts that can fit into the construction platform [38,39]. Ahlhelm et al. combined LCM with Freeze Foaming methods and achieved successful full ceramic structures, combining dense and porous features in a single part of Zirconia suspension. [40] The development of Lithography-based technologies for the industrial production of biomedical parts has been pushed forward with the emergence of solutions such as the CeraFab 7500 (Lithoz, Gmbh, Wien, Austria) and the C3600-ultimate (3DCeram, Limoges, France), the latter based the Lithographic processing by four simultaneous laser sources.

### 1.3. Biological Responses of Nowadays’ Implants

Porous ceramic bone scaffolds and implant coatings allow cell ingrowth and are biocompatible and bioconductive [41]. Calcium sulphate, calcium phosphate and hydroxyapatite are frequently used to manufacture porous ceramic bone scaffolds. Patel et al. structured a scaffold from hydroxyapatite and compared its mechanical behaviour to native bone. The scaffold allows cell attachment and proliferation and it also induced osteoblastic differentiation, which was determined by alkaline phosphatase staining [42]. There is an inverse relation with pore size and mechanical strength of pure porous ceramic bone ceramics. Cell and tissue integration increase with porosity, however, mechanical strength decreases. Li et al. [43] prepared β-TCP microspheres and searched their bioactivity and osteogenic differentiation. They prepared microspheres by the wet precipitation method and characterized them. Later they cultured microspheres with human bone mesenchymal stem cells and checked for cell proliferation using scanning electron microscopy, fluorescent staining, and confocal imaging. RNA (ribonucleic acid) was extracted from the cells cultured with microspheres for 7 days and with quantitative real time PCR (polymerase chain reaction), bone-specific gene expressions were assessed. As a result, microspheres induced the expression of Runt-related transcription factor 2 (RUNX2) and osteocalcin (OCN) specific for β-TCP. Baino et al. [44] searched the in vitro bioactivity of bioactive glass coatings on Alumina/Zirconia composite implants. Bioactive glass coatings were produced either by sponge replication or laser cladding. Once the coated implants were immersed into simulated body fluid, Ca-deficient hydroxyapatite was formed on the surface of the implant. So, the bioactive glass coating triggered the formation of hydroxyapatite due its bioactivity. Coated implants also cultured with human bone marrow derived mesenchymal stem cells and primary osteosarcoma cell lines and cells were proliferated in both lines by time. Sponge replicated samples showed higher mineralization rate than laser cladded samples due to their surface area and porosity. Huang et al. [45] mixed poly-ε-caprolactone with different concentrations of hydroxyapatite or β-TCP and searched for the cell viability and proliferation rates of these composites with human adipose-derived stem cells. According to the results, as the concentration of TCP or hydroxyapatite (HA) increased, cell proliferation rates were increased.

In the study conducted by Abel-Khattab et al. [46], 3D silica containing calcium alkali orthophosphate scaffolds were manufactured by rapid prototyping and compared with scaffolds manufactured by Schwartzwalder Somers method (SSS) according to their mechanical properties and bioactivities. Scaffolds manufactured by rapid prototyping had more porosity than SSS and therefore silica release was much higher. Thus, these scaffolds showed higher cell proliferation and extracellular matrix formation. This also induced the mineralization and osteocalcin expression, which were the signs for osteoinductivity.

In a previous study [47], we demonstrated that intramedullary implantation of porous hydroxyapatite powder implantation may cause bone marrow depletion. This depletion was however due to gas sterilization. We now recommend not to use gas sterilization, or in case we need to use, the application should be considered after 2 weeks of sterilization. Combining polymers and bioceramic powders can be used in 3D printing of porous ceramic bone scaffolds. The quality, type and quantity of the polymer may also determine its biocompatibility [48]. Such composites have recently been used for controlled release of medicines and signalling molecules.

In this study, we ask whether bone spacers produced by green processing of additively manufactured two-layer meshes stimulate (a) mesenchymal stem cell proliferation and adhesion (b) ossification, and (c) improve mesenchymal stem cells osteogenic differentiation. We further ask whether tissue will integrate into the bone spacers produced by green processing of additively manufactured thin formats.

## 2. Materials and Methods

### 2.1. Overview of the Study

The main aim of the present study is to determine and to assess the biological responses yielded by ceramic bone spacers produced by green processing of additively manufactured thin ceramic meshes, in the scope of an international research and development collaborative project (“Origami”: Industrial Manufacturing of Bioceramics by a New High Speed Additive Manufacturing Method under the scheme of collaboration for R&D projects between Spain and Turkey, grant number E!-8053).

The basic geometry for undertaking the biological study is the cylindrical bone spacer defined as the reference work sample in Section 2.2 and the manufacturing process required to manufacture the samples needed is detailed in Section 2.3.The manufacturing process defined consists of an AM processing operation—obtaining bioceramic meshes by robocasting—and the postprocessing operations of rolling, debinding and sintering of the meshes to achieve the desired bone spacer cylindrical geometries.

Once the manufacturing of the samples is completed, the details of the biological testing are presented in Section 2.4. We aimed to present mesenchymal stem cell proliferation, adhesion and Alkaline phosphatase assessment using an ELISA (enzyme-linked immunosorbent assay) reader on days 1, 3 and 7 and DiI fluorescent staining. RT-PCR (real time polymerase chain reaction) was used to quantify COL1A1 (alpha-1 type I collagen), BGLAP (bone gamma-carboxyglutamic acid-containing protein), IBSP (integrin-binding sialoprotein), and SPP1 (secreted phosphoprotein 1) genes relevant to osteogenic differentiation. Subcutaneous implantation aimed to assess tissue compatibility of the manufactured bone spacers.

**Figure 2:**
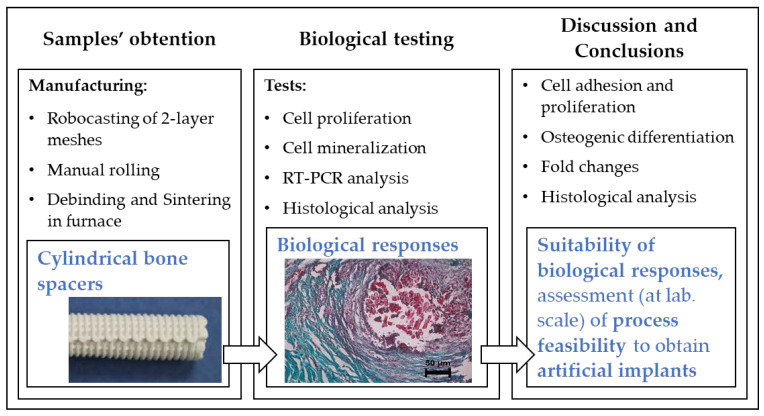
Flow diagram of the study undertaken.

The analysis of cell proliferation, cells differentiations analysis, conveyed the results presented in Section 3 (cell adhesion, osteogenic differentiation, and fold changes). The study finished with the analysis of results in the context of bone spacer implants and the conclusions of the achievements yielded by the study. All these steps are summarized in the flow diagram of the study undertaken that is presented in Figure 2.

### 2.2. Implant Samples Definition and Materials

In the search for a simple implant geometry that could meet the medical and process requirements, a cylindrical bone spacer is identified. To achieve this geometry, the two-layer mesh obtained by robocasting will only need to be rolled in a single operation while in green. As no full bending is required, the geometry is expected to demonstrate better integrity during the postprocessing that could lead to better resistance to crazes.

The material prescribed for the samples was a compound of hydroxyapatite with β-TCP with a ratio 60/40 (wt%), produced as described in [49,50], which proved successful in previous experiences [51]. Hydroxyapatite, that is the inorganic component of the bone, is a biocompatible, biodegradable, osteogenic, and osteoinductive material generally used in bone tissue engineering. Due to its fragile, inelastic nature, hydroxyapatite is generally used with other materials as a composite [52]. Particle Size Distribution (PSD) analysis on the powder material used confirmed a monomodal average particle size of 1–2 μm, with a maximum size below 5 μm. The size distribution of this composition ensured that the aggregate could be injected through nozzle diameters below 850 μm. Also, utilizing this raw material, the green geometries are expected to experiment a contraction by a factor of approximately 30% in size. With this expectation, the diameters of the struts were prescribed relatively big, compared to previous experiences that proved feasible to obtain struts of much smaller diameters. Once the samples are ready for implantation, the biggest pores (peripheral windows) are expected to have dimensions under 400 μm × 400 μm, with smaller pores closer to the driving axis of the cylinder. This morphology is within the values recommended in the literature for bone tissue engineering [11]. The porosity of the part will be connected in the periphery of the implant, allowing the particles of the implantation medium to move axially (in channels) and around the cylinder. The radial direction is expected to present a macroporous staggered strut morphology [53].

### 2.3. Manufacturing of Implant Samples

The green ceramic meshes selected are rectangular grids of 45 × 35 struts to be printed in the flat surface of the robocasting construction platform. The diameter selected for the end of the extrusion syringe is 0.85 mm. The spacing distance is programmed between extrusion lines and it is set to the same size as the extrusion syringe diameter (0.85 mm). The rolling operation is conveyed by rolling in the direction of the shorter side of the rectangle, leading to green cylinders of approximately 75 mm of length and an estimated apparent diameter between 8.5 and 9 mm. Variance may appear depending on the manual force applied during the process. Therefore, it is critical to make sure that during the rolling operation, too much force is not applied that could deform and compact the compound instead of just rolling it. Due to the fact that all struts are separated by the same distance in the plane, once rolled, the disposition of the different rolled mesh’s struts is expected to have some alternate and some blocking struts in the radial direction of the bone spacer. This staggered morphology should not cover all void spaces, but should allow interconnected porosity in the radial direction of the cylinder.

With this sample design, considering the quantity of material deposited and the envelope apparent volume of the sample, the overall porosity in the rolled sample is expected to range between 30%–37%. However, the level of porosity is not expected to be homogeneous in the radial direction. Due to the rolling operation and the heat treatments, the driving axis of the cylinder (central internal part) is expected to be almost full of material while the external perimeter is expected to maintain windows open to the exterior. The porosity level in the periphery (distance of 3 or 4 mm from the perimeter to the driving axis of the cylinder) is expected to range between 67% and 69% of the volume.

The sample geometry described was produced by means of robocasting, rolling and heat treatments. A total of nine samples were needed for the study. The details of each phase in the manufacturing processes can be found in the following sections.

#### 2.3.1. Robocasting of Ceramic Meshes

**Figure 3:**
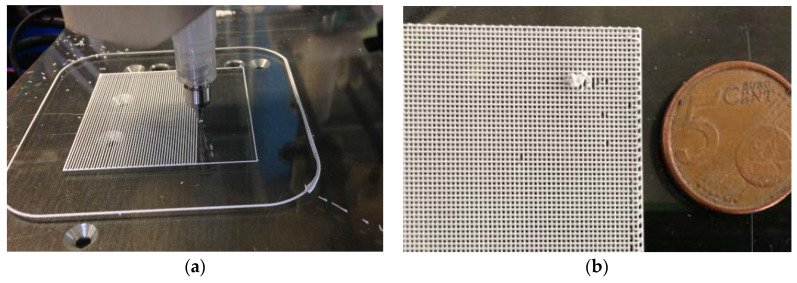
Exemplary images: (**a**) Robocasting system 3D printing the first layer of a squared ceramic mesh; (**b**) Detail of a thin 3D printed ceramic mesh composed of two layers of material.

The robocasting operation for obtaining the thin meshes was performed in a robocasting prototype designed and constructed on purpose by Centre CIM, consisting on a RepRapBCN BCN3D+ FDM machine (Centre CIM, Barcelona, Spain), reconverted with a specific 3D printing head for depositing layers of dense material from a 50 cm^3^ syringe [34]. An example of a similar pattern of a two-layer ceramic mesh (with different total dimensions than the one utilized in the present study) while being printed and after the deposition can be found in Figure 3a,b.

The ceramic meshes deposited for the present study were produced according to the specifications prescribed. The horizontal and vertical separations between extrusion lines were 0.85 mm (same as syringe extrusion end), implying a total expected flat surface of 58.65 mm × 75.65 mm. Although the machine maximum capacity allowed printing several samples at the same time (table size of 230 mm × 160 mm in the cartesian ‘x’ and ‘y’ axis), the decision taken was to manufacture the samples one after another in separate deposition platforms and let them air dry during a day in the laboratory. The deposition speeds were 10 mm/s in each of the axes, which is half of the maximum speeds that the machine can achieve in axis ‘x’ and ‘y’. All the samples utilized in the study did not have discontinuities nor accumulation of material in the deposition process. The samples that encountered defects (such as holes or knots) were discarded from the study. In this respect, operation of introduction of raw material in the syringe was critical to make sure that no bubbles were generated in the deposit and so were ejected by the nozzle head.

#### 2.3.2. Processing of Green Ceramic Meshes into Bone Spacer Shapes

By using AM, it is possible to form 3D green structures by using colloidal concentrations of hydroxyapatite. Even when controlling the ink composition and viscoelasticity properties, the structures generated are very fragile right after the 3D printing process. In this case, it was necessary to let the constructions dry at open air for a few hours to let the sample achieve some minimum consistency to be handled.

Once the ceramic meshes were dried for 1 day in air, a small rolling hand-made operation was performed. The rolling process was performed with the use of a thin PVC film and a 3d-printed ABS tooling. The film also helped to separate the printed ceramic mesh from the construction platform.

#### 2.3.3. Heat Treatments

Following the initial AM processing, the green geometries obtained in the rolling phase were still green and required undertaking further consolidation processes in a furnace prior to proceed to biological testing. The processing of technical green ceramics required the two conventional furnace steps: First, obtaining a brown format (after the debinding step) and then obtaining the final format (after the sintering post processing in forced air ventilation in the furnace) [54].

In general, the sintering temperature is lower than the fusion temperature of the calcium phosphate, and the bonding of the ink particles is due to the processes of diffusion while in solid state at high temperature. Hydroxyapatite is a relatively easy material to sinter at temperatures between 1200 and 1300 °C [28]. However, β-TCP is more difficult to sinter because at temperatures above 1125 °C it experiences an allotropic transformation to the alpha phase [55], meaning that the change in volume could generate crazes in the final part.

The furnace processing of the cylindrical samples included a debinding step and a sintering step. The result of the debinding step was brown parts, which were then sintered to obtain the final samples that were used in the biological testing. The debinding step was performed at a temperature of 600°C for 2.5 h. The sintering step of the brown samples was performed at 1310 °C for 4 h. The parts cooled slowly in the furnace. 

The post-processing conditions imply a profound contraction and distortion of the sintered geometries, that experience a change both in volume and in shape [56]. Adapting the geometrical design and sintering conditions, the structures can be formed with a bi-modal distribution consisting on a meso-structure of big pores between the struts (typically between 200–500 µm) [34] and micropores (1 µm) in the struts [32].

### 2.4. Biological Tests

Human bone marrow originated mesenchymal stem cells (MSC) were purchased (Poietics PT 2501, Lonza, Basel, Switzerland), expanded, and passage 6 was used according to the supplier’s data sheet and literature that used the same cell line [57]. Briefly, cell culture medium consisted of low-glucose Dulbecco’s modified Eagle medium (LG-DMEM; Biochrom AG, Berlin, Germany), along with 10% fetal calf serum (FCS; Biochrom AG, Berlin, Germany) and 1% penicillin/streptomycin (Biochrom AG, Berlin, Germany). The complete medium was replaced every 3 to 4 days. Cells were harvested with 0.25% trypsin/1 mm Ethylenediaminetetraacetic acid (EDTA) and re-plated when adherent cells reached sub-confluence. Passage 6 (P6), mesenchymal stem cells (MSCs) were used for the studies. The material filled almost the entire surface of the culture dish, which remained attached during proliferation, and osteogenic markers. The material group was compared only with cell (blank) group. No osteoblast differentiation factors were used during the experiment to assess the osteoconductivity of the material without any external factor.

#### 2.4.1. Proliferation Test

Cell proliferation assays were undertaken at three different time points (1, 3 and 7 days) in triplicate. Eleven-thousand-seven-hundred-and-nineteen cells/cm^2^ were seeded on each material and control well. MSCs were cultured with a medium that consisted of 52.8% Dulbecco’s Modified Eagle Medium (DMEM) with 1 g/L glucose (Lonza, Basel, Switzerland), 35.2% MCDB-201 medium (Sigma Aldrich, St. Louis, MI, USA), 10% heat inactivated fetal bovine serum (FBS) (Sigma Aldrich, St. Louis, MI, USA), 1% penicillin/streptomycin solution (Biochrom AG, Berlin, Germany), and 1% L-glutamine (Biochrom AG, Berlin, Germany) [42]. The medium was changed every 3 to 4 days. Materials and cells were incubated at 37 °C with relative humidity under an atmosphere of 5% CO_2_. At predetermined time points, the medium was aspirated and 500 µL of fresh medium was added along with 50 µL WST-1 (Roche, Basel, Switzerland) to each well. Culture plates were incubated at 37°C with relative humidity under an atmosphere of 5% CO_2_ for 4 h. After incubation, 110 µL WST-1 containing culture medium was pipetted into a flat bottom 96-well plate and the absorbance of the wells were measured in an ELISA reader (Tecan Sunrise, Mannedorf, Switzerland) at 450 nm with a 620 nm reference wavelength. The statistical significance between control and experimental group was defined with Student *t*-test, and *p* < 0.05 was considered as significant.

Materials and cells were incubated at 37 °C with relative humidity under an atmosphere of 5% CO_2_ for DiI staining and fluorescent microscopy. At day 3, growth medium was removed and replaced with 500 µL staining medium that consists of 5 µL diI stain for each 1 mL of DMEM-LG. The plates were incubated for 20 min at 37 °C and after 20 minutes, staining medium was removed and replaced with serum free growth medium. The cells were incubated with serum free growth medium for 10 min and this washing procedure was repeated three times. Afterwards, the materials were examined by fluorescent microscopy.

#### 2.4.2. Osteoblastic Marker Analysis

Osteogenic potential of the prototypes was evaluated with alkaline phosphatase activity staining. Twenty-three-thousand-four-hundred-and-thirty-eight cells/cm^2^ were seeded on each material and the control wells. MSCs were cultured with proper medium for 21 days at 37 °C with relative humidity under an atmosphere of 5% CO_2_ and the medium was refreshed every 3 to 4 days. No osteoblast differentiation factors were used in medium during the experiment in order to assess the osteoconductivity of the material without any external factor. On day 21, the medium was discarded and 400 µL Alkaline Phosphatase Yellow Liquid substrate system for ELISA (Sigma Aldrich, St. Louis, MI, USA) was added to each well from both groups. The plate was incubated for 30 min, and 100 µL of 3 N sodium hydroxide (NaOH) was added to each well to stop the reaction. Twho-hundred microliters of the final product was pipetted to a flat bottom 96-well plate and analyzed with an ELISA reader (Tecan Sunrise, Mannedorf, Switzerland) at 405 nm wavelength [58,59]. The statistical significance between control and experimental group was defined with Student *t*-test and *p* < 0.05 was considered as significant.

#### 2.4.3. RT-PCR Analysis

For the RT-PCR analysis, human bone marrow originated MSCs (passage 6, total 20,000 cells/cm^2^) were cultured in T75 flasks with cell culture medium and samples at 37 °C with relative humidity under an atmosphere of 5% CO_2_. The medium was refreshed every 3 to 4 days. MSCs were trypsinized with 0.25% Trypsin-EDTA (Invitrogen, Gibco, UK) and suspended in 200 µL PBS after 14 days of incubation. mRNA was isolated using the High Pure RNA Isolation Kit (Roche, Basel, Switzerland). Four-hundred microliters of lysis/-binding buffer was added to the suspended cells and vortexed for 15 s. Samples were pipetted into a filter tube inserted into a collection tube and the entire tube was centrifuged at 8.000× *g* for 15 s. After centrifugation, the liquid in the collection tube was discarded. Ninety microliters of DNase incubation buffer was mixed with 10 µL DNase I and pipetted to the upper reservoir of the filter tube afterwards. Cells were incubated at room temperature for 15 min. Then, they were washed with 500 µL of buffer I that was added to the filter tube and centrifuged at 8000× *g* for 15 s. The liquid in the collection tube was discarded again and this time 200 µL wash buffer II was added to the filter tube and the entire tube was centrifuged at 13,000× *g* for 2 min. The filter tube was removed from the collection tube and inserted into a sterile microcentrifuge tube. Sixty microliters of elution buffer was added to the filter tube and centrifuged at 8000× *g* for 1 min. Complementary DNA (cDNA) was synthesized with Transcriptor High Fidelity cDNA Synthesis kit (Roche, Basel, Switzerland). Total RNA was mixed with 2 µL Random Hexamer Primer and PCR-grade water to make 11.4 µL of total volume. The total RNA quantity from each sample was in the range of supplier’s protocol and homogenously between (500 ng to 1 µg). The tube was incubated at 65 °C for 10 min in Geneamp 9700 Classic PCR machine (Applied Biosystems, Thermo Scientific, Waltham, MA, USA) and immediately cooled on ice. Later, 4 µL transcriptor High Fidelity Reverse Transcriptase Reaction Buffer, 0.5 µL Protector RNase inhibitor, 2 µL Deoxynucleotide mix, 1 µL DTT, and finally 1.1 µL Transcriptor High Fidelity Reverse Transcriptase were added to the tube to make a 20 µL final volume. The reagents were mixed carefully, and the tube was incubated at 29 °C for 10 min and then incubated at 48 °C for 60 min. Finally, reverse transcriptase was inactivated by heating the tube at 85 °C for 5 min. The reaction was stopped by placing the tube on ice and cDNA was stored at −20°C. Real time quantitative PCR analysis was performed using Lightcycler 480 Probes Master Mix (Roche, Basel, Switzerland). Ten microliter Probes Master was mixed with 5 µL PCR grade water; 15 µL PCR mix and 5 µL cDNA were pipetted into each well of the custom plate with specific genes (COL1A1, BGLAP, IBSP, SPP1). Final PCR reaction was conducted in a Lightcycler 480 device with one cycle of pre-incubation (95 °C, 10 min) and 45 cycles of amplification (95 °C, 10 s; 60 °C, 30 s; 72 °C, 1s) and cooling (40 °C, 30 s). Lightcycler 480 software (version 1.5, Roche, Basel, Switzerland) was used to calculate the crossing point (Cp) for target and reference genes with Advance Relative Quantification method. All target genes were normalized to housekeeping genes ACTB (beta actin), GAPDH (glyceraldehyde 3-phosphate dehydrogenase) and G6PD (glucose-6-phosphate dehydrogenase). The results were given as fold change corresponding to mesenchymal stem cells control group according to ΔΔCt calculation.

#### 2.4.4. Subcutaneous Implantation and Histological Analysis

Service was purchased from the Hacettepe University Faculty of Pharmacy, Department of Pharmacology for implantation test. Biological testing was performed according to EN ISO 10993-6: Biological evaluation of medical devices—Part 6: Tests for local effects after implantation Annex A. Experimental samples were cut into three sections with approximately 10 mm of length and these sections were used as implants for the procedure. Briefly, New Zealand rabbits weighing about 500 g were housed in a temperature-controlled room (22–24 °C) with 12-h light and 12-h dark cycles. Rabbits were given free access to water and food without antibiotics for 24 h a day. They were anesthetized by intraperitoneal 0.5 mL ketamine chloride (Ketasol 10%, Richter-Pharma, Wels, Austria) and 0.5 mL xylazine (Alfazyne 2%, Alfasan, Woerden, The Netherlands) injection. An incision was made in the dorsal midline and three subcutaneous pockets were made on both the right and left side by blunt dissection. The base of the pocket was more than 10 mm from the line of incision. Polymethyl methacrylate (PMMA) was used as control material. The total of three experimental implantations and three PMMA implants were implanted to each rabbit and a total of three rabbits were used. Therefore, a total of nine experimental implants were implanted. The skin and subcutaneous tissues were sutured with 2.0 silk (Ethicon, Somerville, NJ, USA), and closure was cleaned and sprayed with an antibacterial film (Opsite, Smith & Nephew, London, UK). Rabbits were put into cages allowing their free movement and fed with regular diet. The animals were sacrificed by lethal doses of anaesthetics after 2 weeks based on EN ISO 10993-6 Section 5.5.3 (a) and implants were removed with surrounding tissue. Formalin fixation was performed to maintain tissue integrity in subcutaneous implant samples obtained from animals. Afterwards, 3–5 micrometre-thick sections were taken from paraffin embedded samples. The sections were deparaffinized in an oven at 60 °C and then made transparent in xylol. Sections passed through 96% and 80% alcohols were stained with Masson Trichrome stain to show routine Haematoxylin & Eosin and collagen. Finally, the mounted sections of a total of six samples were analysed by using a DMR 6000 microscope equipped with the DC500 digital camera (Leica, Wetzlar, Germany) for histological evaluation.

## 3. Results

### 3.1. Production of the Samples Utilising the New Process

The samples were manufactured according to the new process formalized (i.e., robocasting, rolling, debinding, and sintering). The process allowed to obtain nine viable cylindrical spacers for implantation according to the specifications. Considering a machine time for changing from layer one to layer two of 5 s, the total time of the robocasting operation in the new proposed process was 533.7 s (see Table 1).

**Figure 4:**
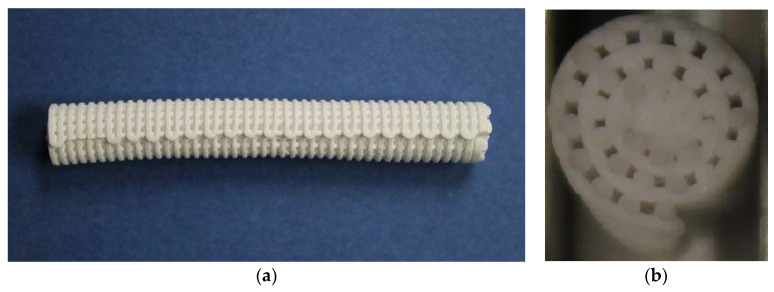
Thin 3D printed mesh of the study once rolled into a bone spacer cylinder shape: (**a**) side view—rolled, in green; (**b**) top view—final part after heat treatments.

The rolling operation requires to be performed with the minimum possible force as the parts are still very fragile (green). Once completed, the processing made the meshes become almost cylindrical rolls. One of the samples obtained is depicted in Figure 4 (side and top views).

**Figure 5:**
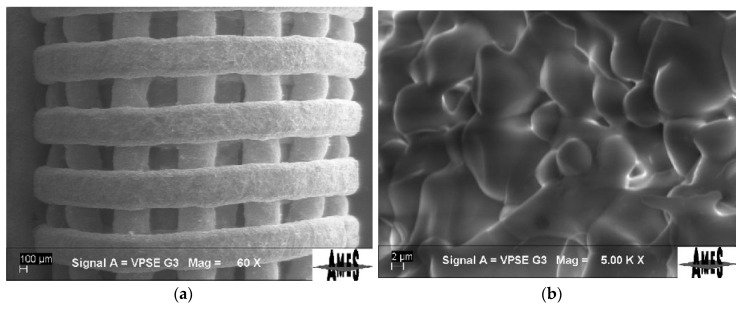
Morphological investigation of a sample prepared for implantation: (**a**) SEM image acquired at low magnification (60×); (**b**) SEM image acquired at high magnification (5000×).

With these parameters, after debinding and sintering, the rolled samples produced reported a maximum 9.5 MPa compression strength. The morphology of the postprocessed samples was observed using Scanning Electron Microscopy (SEM) and showed proper consolidation of the material. The detail of the surface in the samples prepared for implantation is depicted in Figure 5 at low magnification (struts visible at 60×) and high magnification (5000×).

### 3.2. Cell Proliferation

**Figure 6:**
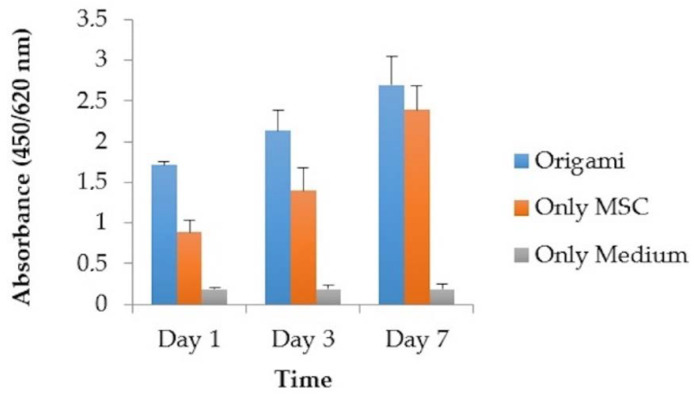
Cell adhesion and proliferation of the material and the control groups (Origami refers to the samples produced in the process addressed in the present article).

The material group had 1.709 ± 0.047, 2.133 ± 0.256 and 2.702 ± 0.344 absorbances for the 1st, 3rd and 7th days, respectively (see Figure 6). The increase in the absorbance values reflected the elevation of cell adhesion and proliferation. The 3D structure of the material enabled rapid cell integration but there is no statistically significant difference between control and experimental groups (*p* = 0.311).

**Figure 7:**
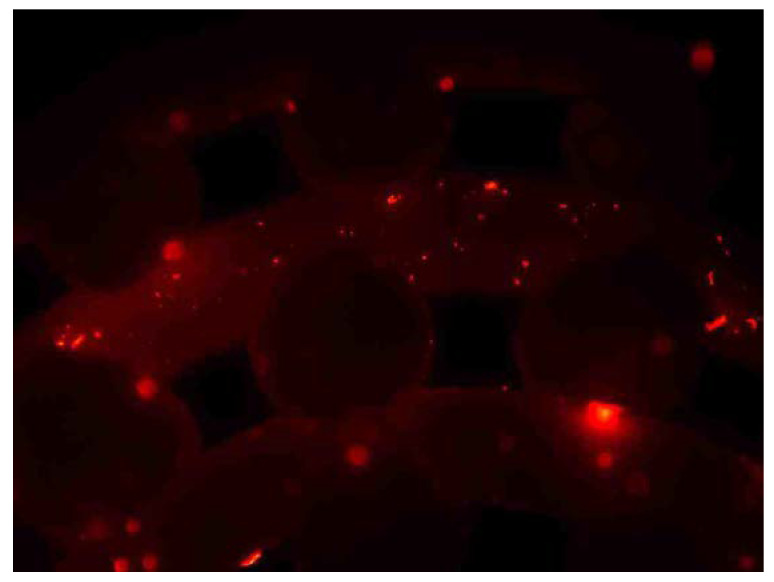
Fluorescent microscopy picture showing the attachment of the cells to the surface of the material.

DiI is a lipophilic membrane stain that diffuses laterally to stain the entire cell. It is weakly fluorescent until incorporated into cell membranes. The pictures obtained from fluorescent microscopy showed that cells were attached to the surface of the material. One example of this is presented in Figure 7.

### 3.3. Osteogenic Activity

**Figure 8:**
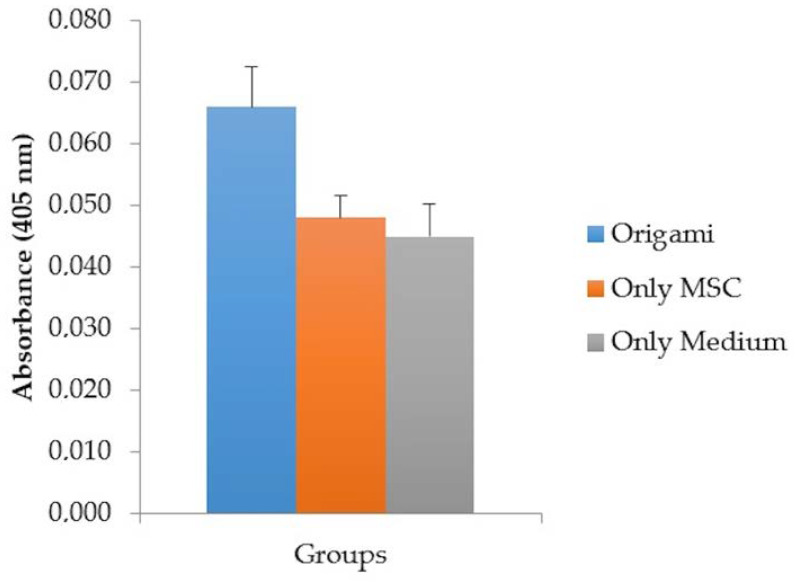
Osteogenic differentiation in the material and the control groups in terms of Alkaline Phosphate Yellow Liquid substrate system absorbance.

After 21 days of incubation, the absorbance for the material group was 0.066 ± 0.007 while it was 0.048 ± 0.004 for the control group (see Figure 8). The bioceramic content in the material triggered the osteogenic differentiation of MSCs and as a result higher absorbance was obtained. There was a statistically significant difference between the control and experimental group (*p* = 0.023), and no significant difference was found between the only MSC and only medium group (*p* = 0.463).

### 3.4. RT-PCR Analysis

**Figure 9:**
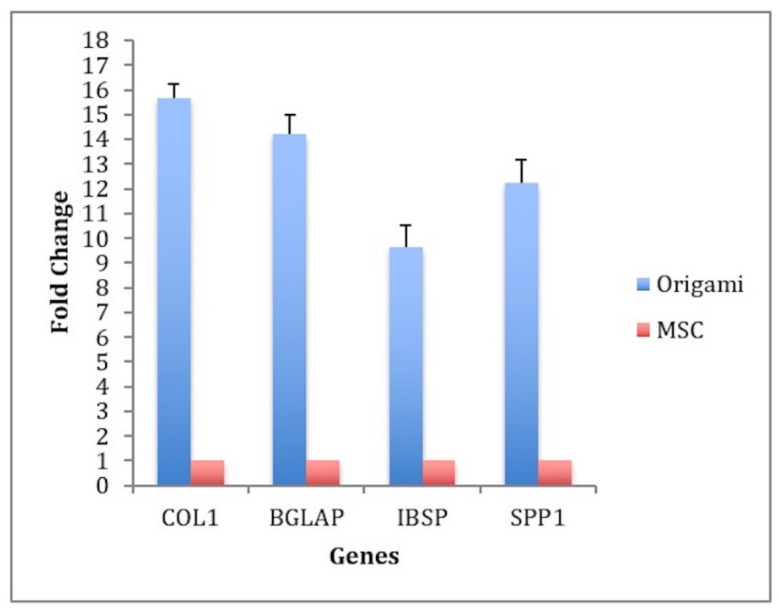
n-fold changes of bone-specific genes in the material and the control groups.

According to the RT-PCR results, COL1A1 gene had 15.66 ± 0.56, BGLAP gene had 14.21 ± 0.76, BSP gene had 9.62 ± 0.89, and SPP1 gene had 12.22 ± 0.96-fold changes in the material group when compared to the control group (see Figure 9). The results showed the osteogenic differentiation of MSCs in the material group along with the osteoconductive characteristic of the material in vitro.

### 3.5. Histological Analysis

**Figure 10:**
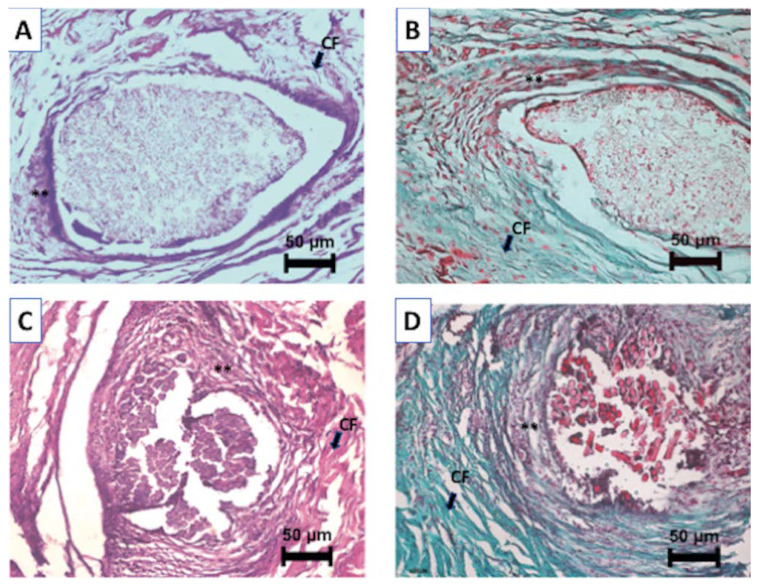
Representative micrographs from the histologic examination of sections: (**A**) implanted with PMMA, Haematoxylin & Eosin 200×; (**B**) implanted with PMMA, Masson’s Trichrome 200× (**C**) implanted with experimental group, Haematoxylin & Eosin 200×; (**D**) implanted with experimental group, Masson’s Trichrome 200×. CF: Collagen fibres, **: Granulation Tissue.

The material implanted to subcutaneous tissue of rabbits triggered a mild granulation tissue and a collagenous tissue formation along with mononuclear cell and fibroblast proliferation. Regarding this, several histological sections of animals showing the implanted areas are presented in Figure 10.

## 4. Discussion

Bone defects around or larger than 10 mm can hardly be replaced by the body. Bone spacers are often used for repairing large bone defects including the cranium, maxillofacial area, spine, and limbs. Considering the constraints imposed when using autografts, allografts, and xenografts, it is worthy to approach the materialization of artificial implants, in particular for large implant sizes.

One of the initial discussion points, which was necessary for addressing the main aim of the article (biological responses), was to successfully achieve the materialization of cylindrical spacer samples utilizing the new process proposed (i.e., robocasting, rolling, debinding, and sintering). Robocasting a ceramic mesh of a hydroxyapatite in combination with β-TCP compound required special attention to prevent discontinuities and accumulation of material in zones of the printed material. Although at laboratory scale this process was complex, it was possible to find a feasible compromise between printing parameters and viable printed meshes.

Over the years, many AM other techniques have been used to fabricate scaffolds. Three of them that have been the object of study and have found consistent results are Stereolithography (SLA) [60], Selective Laser Sintering (SLS) [61], and Fused Deposition Modelling (FDM) [62,63]. In the general case, the manufacturing of basic polymer structures can be completed in any of these three processes, providing that the structure will have to be removed once the ceramic cement is infiltrated. In this respect, SLA restrictions have long been scrutinized, and it exist investigations on ceramic resins yeiding behaviours and properties not affected under the influence of a laser in the normal fabrication process [64]. However, SLA is normally not the preferential option as the polymers it utilizes tend to be difficult to be removed in the following steps.

Concerning the use of SLS for the manufacturing of hydroxyapatite implants, both the particle size of the ceramic powder and the energy density of the laser applied play a fundamental role in the final density achieved [65] as well as to the mechanical properties achieved by the 3D printed part. Some studies prove the efficiency of the SLS for the manufacturing of polymer reinforced compounds with a high content of hydroxyapatite, even in commercially available 3D printing equipment [66]. Also interesting is the use of FDM, as the materials utilized tend to be easily removable by heating without degrading the hydroxyapatite [67]. Koh et al. [68] successfully manufactured structures of poly(ε-caprolactone)/hydroxyapatite (PCL-HA) with FDM. However, processing times in both SLS and FDM tend to be long.

In other cases, the manufacturing of parts controlling the level of porosity can be achieved by indirect procedures, in which the 3D printed structure corresponds to polymer structures in which the ceramic cement (hydroxyapatite) is infiltrated in the pores or cells of such a structure, until the walls of the polymer are entirely covered. Once this is achieved, the polymer is removed, and the new skeleton is strengthened by sintering in a furnace at a relatively high temperature [35]. Indeed, indirect methods can be applied with other geometries than 3D printed, such as sponge replication [69,70]. Still, AM techniques are more attractive as the make it possible to computer design and control the disposition of every bit of the material, rather than copying an established physical pattern.

Concerning post-processing, the rolling operation was performed manually, but it is susceptible to being automated. In the case shown in the Figure 4, a variation in the diameter can be seen along the side view of the roll. This variation is due to a local excess of force applied during the manual rolling operation and demonstrates the fragility of the ceramic mesh when rolling. Also, it is important to mention that the force applied during the rolling has implications on the size of the void spaces achieved between the struts and therefore on the level of porosity and on the continuity of the porosity across the different directions of the spacers. This porosity variation could affect the bone spacer cell activity results. This also indicates room for improvement when conducting this operation by automatic means. Heat treatments were conducted in batch processing (discrete) in furnaces but could also be undertaken in a continuous manner if industrially scaled.

There exists a relationship between the porosity of the implanted material with the cell growth, vascularisation, and nutrient diffusion. Klawitter y Hulbert (1971) [71] established a minimum threshold of 100 µm of porosity to achieve bone growth over the ceramic structure. Itala et al. (2001) [72] proposed that this growth could already be achieved in pore sizes of 50 µm. Tamai et. al. (2002) [73] determined that the interconnection of pores with sizes lower than 10 µm does not enable the migration from pore to pore. Nowadays, a minimum interconnection of 100 µm is accepted, which is necessary for the mineralization of tissues that live on the implants [74]. Therefore, the mesostructure generated with the present process is aligned with the pore interconnection recommended for the colonisation of structures.

Concerning the biological responses yield by the cylindrical bone spacer samples, the porous structure allowed cell adhesion and proliferation [45]. DiI staining qualitatively monitored cells with their intact membranes on top of the sample, and WST-1 revealed quantitatively increased proliferation. Although qualitative evaluation is a limitation, quantitative proliferation data supported the positive effect of our sample on mesenchymal stem cell proliferation. Attached MSCs underwent osteogenic differentiation and we determined this osteogenic differentiation with ALP (Alkaline Phosphatase staining) as an early mineralization marker [58,59,75]. The material slightly stimulated early mineralization when compared to the control assessed using total ALP activity by absorbance values. These findings were in line with the literature on mineralization effect of β-TCP and hydroxyapatite studies [46,58]. The cells on the surface of the bone spacers stimulated bone-specific genes including Col 1, BGLAP, IBSP, and SSP1. The fold change was more than two-fold for these genes. Bone spacers produced by this technology presented mild tissue response when implanted into the back muscles of rabbits. This finding was partially in line with other materials [47] although low or nonimmune reaction could have been established by modifying the components of the bone spacer [48]. As a limitation, osteogenic capacity of the samples can be tested in large-scale animal testing in the future.

At the present time, it has been demonstrated that AM enables to produce engineered constructs to repair or replace damaged tissues [76]. In AM, porous and individually 3D shaped scaffolds can be manufactured for bone cell proliferation, differentiation which leads to bone tissue regeneration [77].

However, the AM processes as methods for direct fabrication for implants are difficult to scale in an industrial way up to mass production in an economically efficient manner and accomplishing all the biomedical quality standards required due to its in-birth discrete nature. For this reason, it has been interesting to address a process modification that conducts robocasting in a way that printing velocity is maximized—by depositing only one layer in the ‘x’ direction and only another one in the ‘y’ direction—and then passing the ceramic meshes obtained to subsequent post-processes.

Concerning the potentiality of the new process to maximize the output in the production of cylindrical spacers, several scenarios can be considered. The time necessary for obtaining the two-layer format by robocasting is 533.7 s; thus constituting the reference scenario.

The first scenario for comparison is the utilization of robocasting to obtain an analogue tall structure of the same external volume (total height of 75.65 mm and diameter of 8 mm) as the samples produced in the present study. The time to print such structure (as it is calculated in Table 2) accounts for 1152 s.

Therefore, in the new proposed method, a time reduction of 618.3 s is encountered in the printing time (before the rolling operation), accounting for 54% of the time required to print the cylindrical structure. The time difference between both alternatives (10.3 min) is more than the time required for rolling the structure with the use of a tooling, thus implying a potential saving in the total time of the two operations (printing plus rolling).

Envisaging the full automation of the process, the conceptualization is that a couple of arrays of printing heads could print the mesh in both directions (‘x’ and ‘y’): Firstly, producing the first layer in a belt-type construction platform with linear moving speed, and secondly producing the second layer moving the second array of printing heads in a perpendicular direction to the belt movement. In this scenario, the printing speed could even be increased, at least to the maximum speed available in the robocasting equipment (20 mm/s). If both material depositions could not be performed synchronously, the total time to print all struts would account for 6.72 s. If the process could be performed in chain, the operation time could even descend to the maximum of both ‘x’ and ‘y’ directions processing time (in this case, 3.78 s from the ‘y’ direction). The detail of this study is presented in Table 3.

Although the industrial scaled up scenario would tend, in principle, to more rigid production, there would still be the chance of obtaining family part variations of the presented implants. For example, the system could adapt to changes in the mesh pattern and to different bone spacer sizes. Additionally, another post-processing cutting operation could be used for selecting and detaching a specific area with a special shape. Finally, in this article, only a rolling operation has been considered, but many other types of processing could be introduced for preparing different implant geometries such as cuboids or others.

Moreover, as a frame of reference of the study, it is interesting to benchmark the process with a very high-speed processing AM process; namely, LCM. In this case, two additional comparison scenarios can be identified with the use of this technology: (a) printing a mesh and (b) printing a full structure. The estimation of the processing times for these are presented in Table 4.

On the one hand, LCM is by far much faster than conventional robocasting and yields much better surface finish and part definition. Even with a layer definition of 11.8% of that used in robocasting (0.1 mm layer height in LCM compared to 0.85 mm in robocasting), the total estimated time to print a full cylindrical geometry via LCM is smaller (1135.5 s). Also, the geometries that can be obtained by LCM can have a high degree of complexity.

On the other hand, the total time estimated to print a mesh by LCM (25.5 s) does not show a significant difference to the total time estimated in the industrial upscaled process. Therefore, it would be interesting to apply the postprocessing operations to the LCM-processed parts and specifically to assess the properties and biological responses achieved.

## 5. Conclusions

The paper aimed at studying and characterizing the biological responses of bone spacers ceramic implants produced by green processing of AM thin meshes. The material used was a compound of hydroxyapatite and β-TCP, that are well-known ceramic biomaterials for bone tissue engineering and show bioactivity, osteoinductivity and osteoconductivity.

Accordingly, cylindrical bone spacers were obtained and biologically tested by several methods; namely: cell adhesion, proliferation, osteogenic differentiation with early mineralization, bone-specific gene modulation, and in vivo response.

Additively manufactured thin bone spacers were feasible to obtain in the new process and stimulated human bone marrow mesenchymal stem cell attachment, proliferation, and differentiation. They also stimulated mineralization. Bone-specific COL1A1, BGAP, BSP, and SPP1 genes changed 8 to 16-fold. These bone spacers were biocompatible when implanted into the subcutaneous tissue of rabbits.

Consequently, the research conducted proved possible to produce functional geometries of hydroxyapatite bone spacers by rolling thin ceramic meshes obtained by AM (robocasting) into cylindrical shapes with continuous open porosity at a lab scale. This process variation is much faster than the normal obtention of parts by conventional robocasting (reporting a production time reduction of 54%), as it avoids the displacements of the nozzle head in the normal direction of the printing plane—normally the ‘z’ axis—which usually accounts for most of the time losses in a 3D printing process.

The process derived is susceptible to be scaled up in terms of output and is easily automatable by means of computer-controlled actuators. Also, more operations such as cutting, bending, and folding could be explored, which could bring more functionalities and versatility to the final parts produced. The discussion also proved that the productivity output (when at an industrial scale) could be aligned even with results yielded by technologies such as LCM, only for the geometry of study, but at the same time benefiting the long range of materials available for robocasting.

Knowing that relatively big bone spacers are almost impossible to harvest from other sources, the presented technology deserves attention for bone spacer production as it could be used in critical conditions. Also, because of the versatility of the fabric-like part production, the presented technology might allow the manufacturers to embrace massive production of bone spacers.

## Figures and Tables

**Table 1:** Case study: Calculation of the total time to print a mesh in the new proposed process.

Parameter	Value
No. Of struts in the ‘x’ direction	45
Length of the struts in the ‘x’ direction	58.65 mm
Total length to be printed in the ‘x’ direction	2639.25 mm
No. Of struts in the ‘y’ direction	35
Length of the struts in the ‘y’ direction	75.65 mm
Total length to be printed in the ‘y’ direction	2647.75 mm
Total length to be printed	5287 mm
Printing speed	10 mm/s
Total time to print the struts in ‘x’ and ‘y’	528.7 s
Time for changing from one layer to another	5 s
Total time to print a sample	533.7 s

**Table 2:** First scenario for comparison (conventional robocasting): calculation of the total time to print an analogous tall sample (cylindrical spacer of the same external dimensions) in robocasting of multiple layers.

Parameter	Value
Total spacer height	75.65 mm
Layer definition (layer height)	0.85 mm
Number of layers required	89
Printing time per layer	8 s
Time for changing from one layer to another	5 s
Total time to print a sample	1152 s

**Table 3:** Second scenario for comparison (industrial upscale): Calculation of the total time to print a mesh in the new proposed process once industrially upscaled.

Parameter	Value
Length of the struts in the ‘x’ direction	58.65 mm
Printing speed in the ‘x’ direction	20 mm/s
Total time to print the ‘x’ direction struts	2.93 s
Length of the struts in the ‘y’ direction	75.65 mm
Printing speed in the ‘y’ direction	20 mm/s
Total time to print the ‘y’ direction struts	3.78 s
Total time to print all struts if not synchronous	6.72 s

**Table 4:** Third and fourth scenarios for comparison: Estimation of the total time to print utilizing LCM technology: (a) values for printing a mesh; (b) values for printing a full structure of equivalent height.

Parameter	Value for mesh (a)	Value for full structure (b)
Total height	1.7 mm	75.65 mm
Layer definition (layer height)	0.1 mm	0.1 mm
Number of layers needed	17	757
Total time to consolidate a layer	1.5 s	1.5 s
Total time to print the geometry	25.5 s	1135.5 s
